# Supplementary material for: The Prebiotic Activity of a Novel Polysaccharide Extracted from Huangshui by Fecal Fermentation In Vitro
Source: Foods. 2023 Dec 7;12(24):4406. doi: 10.3390/foods12244406 (PMC10743195; doi:10.3390/foods12244406)
Supplement: Supplementary file 1 [file foods-12-04406-s001.zip › foods-2741777-supplementary.pdf]

The prebiotic activity of a novel polysaccharide extracted from *Huangshui* by fecal fermentation *in vitro*

Mei Li[1], Jian Su[2], Jihong Wu[1*], Dong Zhao[2], Mingquan Huang[1], Yanping Lu[2], Jia Zheng[2], and Hehe Li[1]

[1] Key Laboratory of Brewing Molecular Engineering of China Light Industry, Beijing Technology and Business University, Beijing 100048, China
[2] Key Laboratory of Soild-state Fermentation and Resource Utilization of Sichuan Province/Key Laboratory of Strong Flavor Baijiu Soild-state Fermentation of China Light Industry/Engineering Technology Research Center of Baijiu Brewing Special Grain of China, Wuliangye Yibin Co. Ltd., Yibin 644007, China
* Correspondence: wujihong12@126.com; Tel.: +86-156-5271-2036

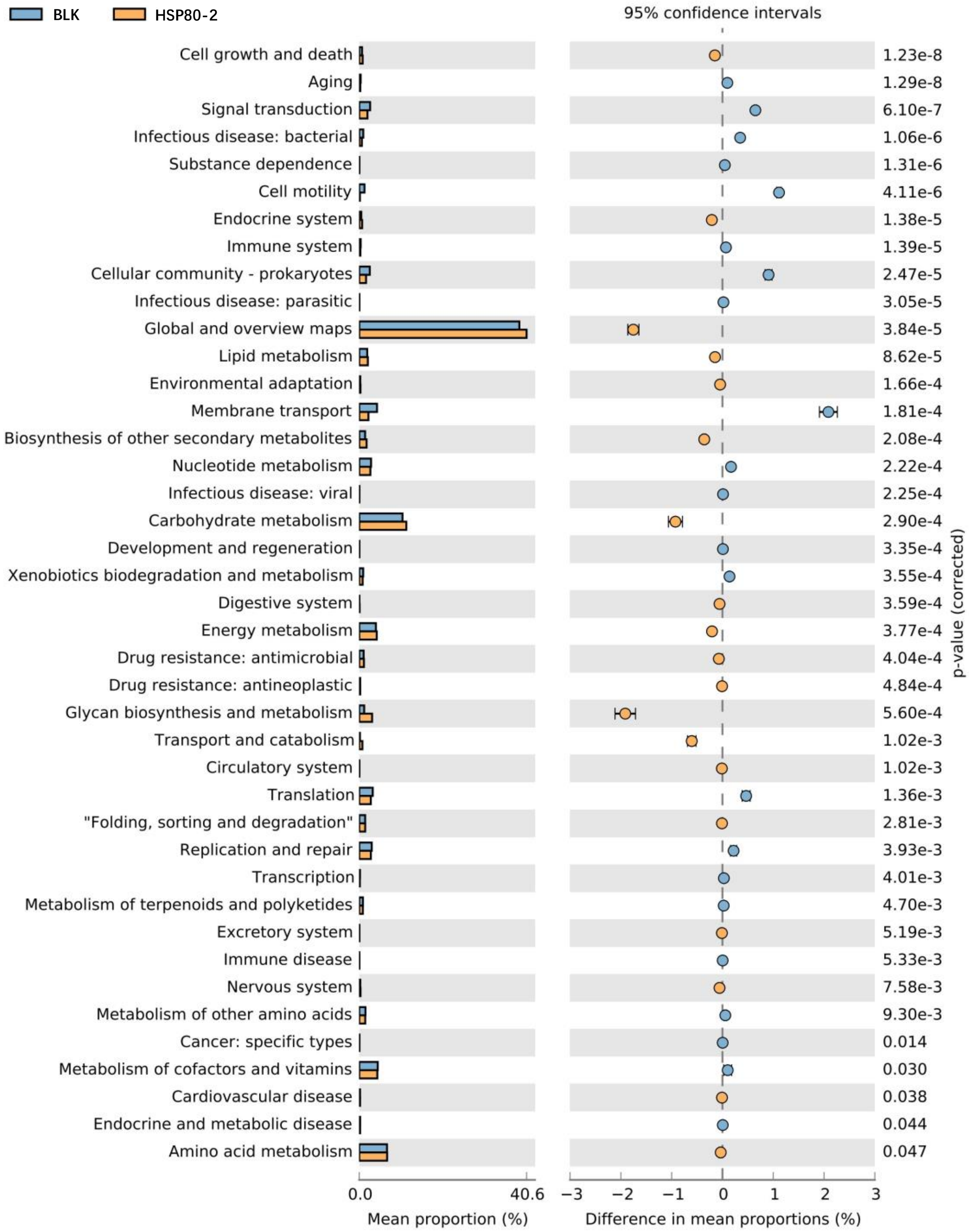

Figure S1 41 significantly different KEEG pathways on level 2

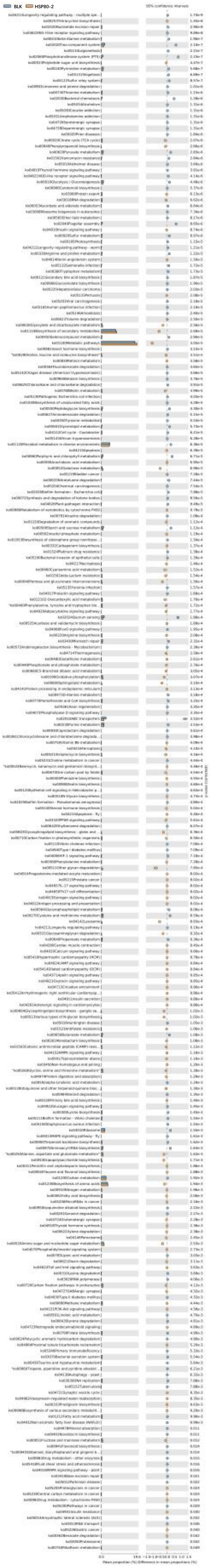

Figure S2 256 significantly different KEEG pathways on level 3
